# Are motivational and self-regulation factors associated with 12 months’ weight regain prevention in the NoHoW study? An analysis of European adults

**DOI:** 10.1186/s12966-023-01529-8

**Published:** 2023-10-27

**Authors:** António L. Palmeira, Marta M. Marques, David Sánchez-Oliva, Jorge Encantado, Inês Santos, Cristiana Duarte, Marcela Matos, Almudena Carneiro-Barrera, Sofus C. Larsen, Graham Horgan, Falko F. Sniehotta, Pedro J. Teixeira, R. James Stubbs, Berit L. Heitmann

**Affiliations:** 1 CIDEFES, Universidade Lusófona, Campo Grande, 376, 1749-024 Lisbon, Portugal; 2 Comprehensive Health Research Centre (CHRC), NOVA Medical School | Faculdade de Ciências Médicas, Universidade NOVA de Lisboa, Lisbon, Portugal; 3 Department of Didactics of Musical, Plastic and Body Expression, Faculty of Sports Sciences, University of Extremadura, Cáceres, Spain; 4 CIPER-FMH, University of Lisbon, Lisbon, Portugal; 5 Laboratório de Nutrição, Faculdade de Medicina, Centro Académico de Medicina de Lisboa, Universidade de Lisboa, Lisbon, Portugal; 6 Instituto de Saúde Ambiental (ISAMB), Faculdade de Medicina, Universidade de Lisboa, Lisbon, Portugal; 7 Appetite Control and Energy Balance Group, School of Psychology, University of Leeds, Leeds, U.K.; 8 Center for Research in Neuropsychology and Cognitive-Behavioural Intervention (CINEICC), University of Coimbra, Coimbra, Portugal; 9 Department of Psychology, Universidad Loyola Andalucía, Seville, Spain; 10 Research Unit for Dietary Studies, The Parker Institute, Bispebjerg and Frederiksberg Hospital, The Capital Region, Copenhagen, Denmark; 11 Biomathematics & Statistics Scotland, Aberdeen, U.K.; 12 Department of Public Health, Social and Preventive Medicine, Center for Preventive Medicine and Digital Health (CPD), Mannheim Medical Faculty, University of Heidelberg, Mannheim, Germany; 13 Department of Public Health, Section for General Medicine, University of Copenhagen, Copenhagen, Denmark

**Keywords:** Weight regain prevention, Motivation, Self-regulation, Mediation

## Abstract

**Purpose:**

Preventing weight regain can only be achieved by sustained changes in energy balance-related behaviors that are associated with weight, such as diet and physical activity. Changes in motivation and self-regulatory skills can support long-term behavioral changes in the context of weight loss maintenance. We propose that experiencing a supportive climate care is associated with enhanced satisfaction of basic psychological needs, intrinsic goals, and autonomous motivation. These factors are expected to be associate with the utilization of self-regulation skills, leading to more sustained behavior changes and ultimately preventing weight regain. This hypothesis was tested in this ancillary analysis of the NoHoW trial, where the study arms were pooled and followed for 12 months.

**Methods:**

The NoHoW was a three-center, large-scale weight regain prevention full factorial trial. In this longitudinal study, data were collected in adults who lost > 5% weight in the past year (*N* = 870, complete data only, 68.7% female, 44.10 ± 11.86 years, 84.47 ± 17.03 kg) during their participation in a 12-month digital behavior change intervention. Weight and validated measures of motivational- and self-regulatory skills-related variables were collected at baseline, six- and 12 months. Change variables were used in Mplus’ path analytical models informed by NoHoW’s logic model.

**Results:**

The bivariate correlations confirmed key mediators’ potential effect on weight outcomes in the expected causal direction. The primary analysis showed that a quarter of the variance (*r*2 = 23.5%) of weight regain prevention was achieved via the mechanisms of action predicted in the logic model. Specifically, our results show that supportive climate care is associated with needs satisfaction and intrinsic goal content leading to better weight regain prevention via improvements in self-regulatory skills and exercise-controlled motivation. The secondary analysis showed that more mechanisms of action are significant in participants who regained or maintained their weight.

**Conclusions:**

These results contribute to a better understanding of the mechanisms of action leading to behavior change in weight regain prevention. The most successful participants used only a few intrinsic motivation-related mechanisms of action, suggesting that habits may have been learned. While developing a digital behavior change intervention, researchers and practitioners should consider creating supportive climate care to improve needs satisfaction and intrinsic goal contents.

**Trial registration:**

ISRCTN, ISRCTN88405328, registered 12/22/2016.

**Supplementary Information:**

The online version contains supplementary material available at 10.1186/s12966-023-01529-8.

## Background

Understanding and preventing weight regain in individuals who successfully lost weight is crucial from a public health perspective. However, while extensive evidence exists about the role of energy-balance related behaviors – physical activity and healthy eating—for sustained weight management [1], less is known about the individual factors influencing these behaviors. The scope of this study will focus on factors associated with motivation [2] and self-regulation [3] as putative predictors of weight management-related behaviors and consequently on weight maintenance outcomes.

Concerning motivation, there is a body of research examining the effects of the satisfaction of psychological needs and autonomous vs. controlled motivations derived from the Self-Determination Theory—SDT [4]. In summary, these studies show that higher levels of needs satisfaction and autonomous forms of motivation are associated with sustained behavioral efforts and better weight regain prevention [2, 5]. Furthermore, these findings are particularly evident in the long-term, where autonomous motivation exerted a positive effect on medium and long-term weight control (≥ 12 months) on all eight times it was tested via mediational analysis [3].

Higher needs satisfaction occurs when the context is perceived as need-supportive, providing autonomy support, structure, and involvement. In these contexts, behavior regulation becomes internalized and integrated, a path leading to increased motivational quality (i.e., autonomous motivation). The SDT postulates that autonomous motivation is the most sustainable form of energizing and directing one’s behavior (which can be branded as “wantivation”). Conversely, in needs frustration contexts, behavioral regulations become externalized, leading to controlled motivations that are less sustainable (which can be branded as “mustivation”), as they only energize and direct behavior as long as external contingencies remain present (Ryan & Deci, 2000).

Concerning self-regulation, a systematic review showed that self-regulatory skills use (such as self-monitoring or goal setting and planning) is a mechanism of action exerting a positive effect on short-term weight control (< 12 months) in 92% of the times it was tested, and, in the medium to long-term (≥ 12 months), 83% of the times it was tested [3]. In the present study, we focused on three dimensions of self-regulation: action planning, action control, and coping planning. Action planning involves developing plans specifying where, when and how goal-directed behaviors link with specific environmental cues. An example of an action plan is, “When arriving at my job, I will park the car further and walk 10 min to work”. This process reduces the resources needed to engage in the desired behavior. Action control refers to, for example, awareness of standards and self-monitoring efforts that enable individuals to attain their goals, even in the presence of competing action tendencies. Finally, coping planning anticipates potential risk situations, linking them to specific behavioral responses. In this sense, it reduces the resources needed to engage in the coping behavior. Examples of coping plans are “IF, THEN” cognitions, such as “If I had planned to have a healthy meal, but I’m starving, I will immediately eat a soup” [6, 7].

Notwithstanding, most of the evidence is focused on how these putative predictors affect behavior change initiation, i.e., where participants are invited to a trial to start doing something new. Even the available long-term data mainly focuses on why and how participants learn and then maintain newly discovered processes. Instead, the present study looks mostly at behavior maintenance processes [8], as participants were asked to start the trial after a successful weight loss period (> 5% weight loss in the past 12 months), and the intervention empowered them to maintain the weight management behaviors used during their weight loss phase. Therefore, we studied predictors of behavior maintenance related to two theoretical themes that Kwasnicka and colleagues identified: i) maintenance motives; and ii) self-regulation. Maintenance motives represent the tendency to maintain behavior due to sustained motives such as satisfaction with the behavioral outcomes, enjoyment, or when the behavior is aligned with values and beliefs – hence linked with the SDT. The self-regulation theme focuses on how people tend to sustain their behavior by successfully self-monitoring and regulating their actions and can engage in effective strategies to overcome difficulties – hence related to self-regulatory processes [8].

These two themes integrate mechanisms of action – the processes through which interventions bring about change—which are worked via behavior change techniques (BCTs) included in the intervention [9]. The present paper is based on a large-scale digital behavior change intervention aimed at weight loss maintenance – the NoHoW study [10]. In the NoHoW logic model, mechanisms of action such as autonomous motivation and action plans are hypothesized as mediators of intervention content (BCTs / active ingredients, e.g., autonomy support) and the intervention outcomes (i.e., weight change).

Notwithstanding, the NoHoW logic model covers only two behavior maintenance themes suggested by Kwasnicka et al. According to that paper, resources, environmental and social influences, and habits also play a role in long-term weight regain management [8]. Of particular interest for the NoHoW intervention, habit development, and dual-process models should be considered [11]. In the dual-process models, there is a “fast” system 1 corresponding to the intuitive and emotional processes and a “slow” system 2 corresponding to the rational and logical operations. Haidt [12] suggested a helpful metaphor for the dual-process model – where system 1 is an elephant (sub-conscious, where habits live), and system 2 is the elephant rider (conscious, where habits development occurs). Hence, we expect the rider to work harder while looking for the initial solutions for a behavior change challenge – a conscious stage. As behavior regulation becomes internalized, more automatic, habits-related, system 1 / sub-conscious processes are expected to emerge as the elephant takes over and uses the learned habits to regulate our behaviors [13].

Most of the evidence above, about the effects of self-regulatory and motivational-based interventions, was developed in face-to-face settings, which are challenging to scale up in a problem as prevalent as weight loss maintenance. The use of digital-based behavior change interventions (DBCI), which include digital versions of the contents used in face-to-face interventions, is expected to provide a scalable and sustainable solution to change obesity-related behaviors [14]. But the evidence on the effectiveness of DBCIs is still scarce. In one of the first reviews on DBCIs, Webb et al. showed that interventions using a larger number of BCTs, framed in a theory base-intervention, had larger positive effects on health-related behaviors [15]. However, more recent reviews focusing on overweight and obesity settings show that DBCIs have small effects [16] and present high variability in the methods and outcomes used [17]. Another review focused on the effectiveness of BCTs in promoting Physical Activity in overweight or obesity settings showed a pooled effect size for digital interventions of 0.42 (range -0.10 to 3.34). Furthermore, the meta-regression showed that goal-setting and social incentives were the most effective BCTs [18].

These results are similar to Webb et al. review in that DBCIs for weight management are more effective than minimal interventions and that using extra components (i.e., follow-up telephone calls) is associated with better outcomes. Notably, while these papers reviewed the interventions’ components’ quantity and mode of delivery alongside a brief description of the materials (e.g., self-monitoring and educational contents), they did not analyze the intervention’s component’s quality—which BCTs and how they were associated with the mechanisms of action and the programs’ effectiveness (the exception is the Carraça et al., 2021 paper). Indeed, a 2022 scoping review showed that only one of the included studies described all the putative links between all the BCTs and the mechanisms of action, while five only partially explained these links [19].

In the paper describing all the links, Sniehotta et al. examined the effectiveness of a low-intensity DBCI to support weight loss maintenance in 288 adults that have lost ≥ 5% of their weight – the NULevel study [20]. The 6-month intervention consisted of a single face-to-face induction meeting, followed by regular tailored and automated SMS about their progress or directing them to educational content. In addition, participants were asked to do daily weighings and access an online study interface to visualize their progress, record and comment on their weekly results, or request further assistance. Albeit no differences in weight (the primary outcome) were detected at the 12-month’ follow-up between the experimental and a minimal intervention control group, the authors observed significant changes in several mechanisms of action. For example, the experimental group reported more planning, greater satisfaction with the outcomes, and greater confidence in engaging in healthy behaviors. We could not find published reports about if the positive changes in the mechanisms of action were associated with the primary or secondary outcomes of the NULevel.

The studies reviewed above are examples of evidence scarcity about how the putative mechanisms of action targeted by DBCI’s contents affect the outcomes (i.e., via mediational analysis). This scarcity may be related to the null-to-small effects commonly observed in weight management DBCIs. Notwithstanding, we agree with O’Rourke and MacKinnon when they suggest doing mediational analysis even in the absence of intervention effects for the valuable information it may provide about the intervention successes or failures [21]. In addition, there is considerable variability in weight results during the weight maintenance period. Previous research [22] has categorized this variability, but little is known about how potential psychosocial factors contribute to weight change in these different groups or how they may be associated with the actual weight change. To address this gap in knowledge, we have developed a secondary aim for this study. By utilizing our large sample size, we aim to conduct complex analyses within three sub-groups that were suggested previously, in order to understand how these factors may be associated with weight change outcomes.

**Fig. 1:**
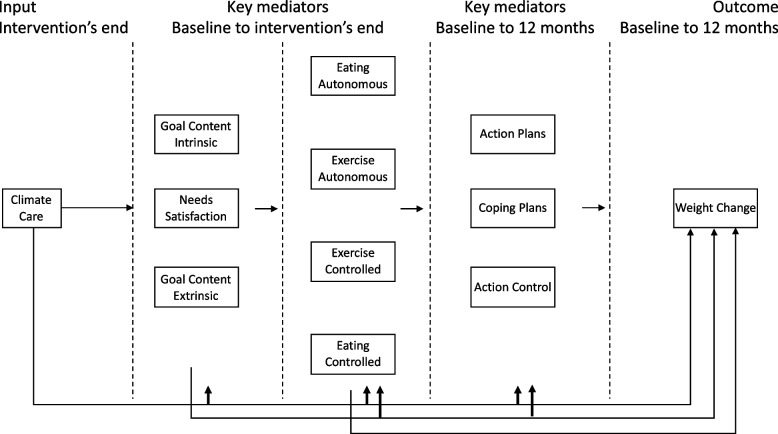
Operationalization of the NoHoW logic model for the present study

In summary, the recent area of DBCI in weight management contexts is evolving rapidly. Recent reviews point to the importance of testing trials with evidence- and theory-based interventions [23, 24], assessing the fit of their logic models to the collected data while depicting the putative links between mechanisms of action and outcomes. While this study does not analyze the mechanisms of action, its results aim to fill this gap by providing information on the variable associations of the model throughout the 12-month trial. It considers two behavior change theories linked to motivation and self-regulation for sustained weight management, hypothesizing that an intervention with BCTs fostering the basic psychological needs, intrinsic goals, and autonomous motivation for exercise and eating could be associated with increased and better use of behavior self-regulatory skills, which, in turn, will promote sustainable healthy behaviors and subsequent weight regain prevention. These assumptions are depicted in the present study’s Logic Model (Fig. 1). This theory-driven model was informed by the NoHoW logic model – a proof-of-concept trial of a DBCI for weight regain prevention. In this context, this ancillary study of the NoHoW trial sought to determine if a logic model informed by self-determination theory and self-regulation rationales could be linked to body weight change for 12 months. Specifically, we hypothesized that a supportive climate care would be linked to needs satisfaction, intrinsic goals, and autonomous motivation, which, in turn, would be linked to self-regulation processes associated with better weight management results. In addition, we hypothesized that the significant associations patterns would differ in sub-groups with different weight change results.

## Methods

### Study design

The NoHoW trial is a three-centre (University of Leeds (UK), The Parker Institute (Denmark) and University of Lisbon (Portugal)) 2 × 2 factorial, randomized, single-blind, controlled trial testing the proof-of-concept of a digital toolkit for weight regain prevention in over 1600 participants (European Union’s Horizon 2020 research and innovation program under grant agreement No. 643309).

The intervention was developed according to the Medical Research Council Framework for developing and evaluating RCTs for Complex Interventions to Improve Health [25]. More details of the trial were published elsewhere [10], and the trial was registered with the ISRCTN registry (ISRCTN88405328). The study was conducted in accordance with the Helsinki Declaration. Ethical approval was granted by local institutional ethics committees at the University of Leeds (17–0082; February 27, 2017), the University of Lisbon (17/2016; February 20, 2017), and the Capital Region of Denmark (H-16030495; March 8, 2017).

For this ancillary NoHoW study, baseline, 6-month, and 12-month data from the four experimental arms were pooled into one single group, as such, the present study is observational and longitudinal in nature.

### Participants

Individuals were eligible if they were aged 18 years or older, had verification of > 5% weight loss in the 12 months before recruitment (excluding surgical weight loss), and had a BMI of > 25 kg/m2 before weight loss. In total, 1627 participants enrolled in the NoHoW study (68.7% female, 44.10 ± 11.86 years, 84.47 ± 17.03 kg). For the present analysis, and because the analytical models need complete data of all variables, the sample comprised 870 participants (67.0% female, 45.71 ± 11.40 years, 83.47 ± 16.19 kg). The sample evenly included participants from all intervention groups and countries.

In comparing the baseline values of the variables of interest between the sub-sample and the total sample, we found that the sub-sample exhibited higher values in autonomous behavioral regulations for exercise, basic psychological needs satisfaction, and goal contents related to weight management (specifically in the health dimension). Additionally, the sub-sample showed lower values in weight and goal contents for weight management (specifically in the image dimension). However, it is important to note that all observed differences had small effect sizes (Cohen's d equal to or lower than 0.170).

### Intervention

Note that, for this particular study, the sample was pooled in just one group. This decision is based on the fact that there were no differences between the intervention arms (the main outcomes paper is under review). As such the description below is to inform of the overall format and content of the intervention; this ancillary study will not compare the impact of the different intervention contents, thus it is observational in nature.

The NoHoW study was a digital behavior change, evidence-based, intervention. In summary, participants were randomized to one of four groups that accessed different digital contents to develop weight regain prevention behaviors: the NoHoW Toolkit. The four groups of the 2 × 2 factorial design were: 1) control; 2) motivation and self-regulation; 3) emotion regulation; and 4) motivation and self-regulation plus emotion regulation.

In addition, all participants received commercial wireless body weight scales (Fitbit Aria), activity trackers (Fitbit Charge 2), and had access to a dashboard with physical activity, sleep, and weight data, which allowed for visualizing long-term progress and enabled simple self-assessments of mood and satisfaction with diet, sleep, activity, and weight as star ratings (1 to 5 stars), and entering free text personal notes into a diary.

The participants in the three intervention arms received intervention content in the form of weekly sessions displayed in the Toolkit as an interactive map. The motivation and self-regulation arm had 17 sessions, the emotion regulation arm also had 17 sessions, and the combined arm had 34 sessions. Intervention participants were encouraged to complete the intervention sessions during the first 18 weeks of the trial. This was achieved by sending participants weekly emails during this time to introduce the weekly themes and remind them to visit the Toolkit. The control arm also received weekly emails for the first 18 weeks, but they only contained links to generic weight management content. A detailed description of the Toolkit is presented by Marques et al. [26].

### Measures

Data collection was conducted at baseline, six, and 12 months. The exception was the Virtual Climate Care Questionnaire, collected once at six months, corresponding to the intervention’s end.

The questionnaires used in the trial were adapted and validated for Danish, Portuguese, and English languages.

#### Virtual climate care questionnaire

Treatment autonomy support was assessed using the Virtual Care Climate Questionnaire – VCCQ [27]. The VCCQ was adapted from the original Health Care Climate Questionnaire [28] and measures perceived autonomy-support in a virtual care setting (i.e., digital intervention). Items capture the participants’ interaction with a digital Toolkit (e.g., “NoHoW Toolkit answers my questions fully and carefully”) instead of a face-to-face setting. The original scale has 23 items with a score range of seven points on a Likert scale (1-strongly disagree; 7-strongly agree); higher scores representing higher levels of perceived support for autonomy from the NoHoW Toolkit. Because some features assessed in the original scale were not included in the NoHoW toolkit, three specific items were removed (e.g., “ < name intervention > takes into account my emotions in the advice given”). VCCQ demonstrated good reliability (McDonalds’ Omega = 0.95).

#### Basic psychological needs satisfaction

The satisfaction of the basic psychological needs was measured by the Basic Psychological Needs Satisfaction Scale—BPNSS [29], adapted to the weight management context, by adding “weight management” to the stem and some specific items. The scale consists of 16 items distributed in three dimensions, one for each basic psychological needs (i.e., Autonomy, Competence, Relatedness). To the intent of this study, a global score was computed by averaging all items’ responses to express overall basic psychological needs satisfaction. Scoring ranged from one to seven points Likert scale (1-strongly disagree; 7-strongly agree) and higher scores represent increased need satisfaction. The BPNSS for Weight Management demonstrated good reliability for all time points (see the Supplemental – Table 1).

Note that we will refer to this variable as needs satisfaction in the results section to improve readability.

#### Goal content for weight management

This construct was assessed by the Goal Content for Weight Management Scale – GCWMS –, adapted from previous work regarding exercise [30, 31]. Adaptations for the weight management context were made by adding “I manage my weight” to the stem and specific items. The GCWMS comprises sixteen items arranged in four subscales (Health Management; Challenge; Social Recognition; Image). Two theoretical dimensions were computed based on the SDT framework to discriminate between intrinsic goals (Health Management; Challenge) and extrinsic goals (Social Recognition; Image). Likert scale scoring ranged from one to seven points (1-strongly disagree; 7-strongly agree); higher scores represented increased intrinsic or extrinsic goals for weight management. The Goal Content for Weight Management Scale demonstrated good reliability for all time points (see the Supplemental – Table 1). In addition, convergent and divergent validity and the multi-group invariance test demonstrated strong measurement invariance between genders [32].

#### Regulations for eating behavior

The regulations for eating behavior were assessed by the Regulations for Eating Behaviour Scale – REBS – [33]. The scale comprises 24 items distributed across six dimensions: Intrinsic, Integrated, Identified, Introjected, External, and Amotivation. Likert scale scoring ranged from one to seven points (1 – not true for me; 7 – very true for me), and higher scores represent a higher manifestation of behavioral regulation. One item was removed from each subscale to reduce filling time, totaling 18 items. The lowest factor loading from an extensive database in an international study was the criteria used for the item removal [34]. This reduced version demonstrated good reliability for all subscales in all measured moments (see the Supplemental – Table 1). In addition, two theoretical SDT-based dimensions were computed, representing good-quality motivation (Autonomous Motivation that includes intrinsic, integrated, and identified regulations) and low-quality motivation (Controlled Motivation that includes introjected and external regulations).

#### Behavioral regulations for exercise

The behavioral regulations for exercise were assessed by the Behavioral Regulations for Exercise Questionnaire – BREQ-3 – [35, 36]. This scale comprises 24 items distributed into six subscales: Intrinsic, Integrated, Identified, Introjected, External, and Amotivation. Likert scale scoring ranges from one to seven points (1 – not true for me; 7 – very true for me), and higher scores represent a higher manifestation of a self-determined behavioral regulation. The BREQ demonstrated good reliability for all time points (see the Supplemental – Table 1). In addition, two theoretical dimensions were computed based on the SDT framework to discriminate between good-quality motivation (Autonomous Motivation that includes intrinsic, integrated, and identified regulations) and low-quality motivation (Controlled Motivation that includes introjected and external regulations).

#### Self-regulation variables

##### Action planning and coping planning

The adapted Action Planning and Coping Planning Scales [37] to the weight management context assessed the self-regulatory capacities for weight management by adding “To manage my body weight” to the stem and specific items. The Action Planning scale comprises four items, and the Coping Planning scale contains 15 items. Scoring ranged from one to five points Likert scale (1-strongly disagree; 5-strongly agree), and higher scores represent higher self-regulatory skills for weight management. The Action Planning and the Coping Planning Scales for Weight Management demonstrated good reliability for all time points (see the Supplemental – Table 1).

##### Action control scale

An adapted Action Control Scale assessed action control based on previous work regarding exercise [37]. Adaptations for the weight management context were made by adding “weight management” to the stem and specific items. The Action Control scale comprises eight items in three subscales: self-monitoring, awareness of standards, and self-regulatory effort. Scoring ranged from one to five points Likert scale (1-strongly disagree; 5-strongly agree), and higher scores represent higher action control for weight management. The Action Control Scale for Weight Management demonstrated good reliability for all time points (see the Supplemental File – Table 1).

#### Outcome measure

##### Objectively measured weight

Body weight was measured at each clinic visit (at baseline, 6 and 12 months), with participants wearing light clothes using a Seca 704 s instrument (SECA, Germany).

### Statistical analysis

The NoHoW project tested a model informed by the NoHoW with two paths: one representing motivational plus self-regulation processes; and another representing emotional and stress regulation processes. The present study focused on the first path, depicted in Fig. 1. Specifically, we are looking at how the climate care of the intervention (i.e., input) interacted with the motivational and self-regulation mechanisms of action (i.e., key mediators) to affect body weight change as the study’s primary outcome. The overall logic model included extra paths related to emotional regulation variables that are not being considered in the present study. This decision was based on the complexity of the analytical procedures encompassing more than 20 variables and on the NoHoW publication plan that divided the research questions based on the partners expertise.

We used change scores of the variables in the correlational and mediational analysis. These variables were expressed by the residuals of the 6-month or 12-month value regressed on the baseline score. Using such residualized change scores is recommended as it creates a value orthogonal to the pre-treatment value(s) and represents a preferable measure of change compared with the pre-post subtraction procedure [38]. According to the model, we calculated the 0–6 months residuals for the motivation-related variables – representing the intervention period – and the 0–12 months residuals for the self-regulation related variables to represent both the expected effect of the intervention and the motivation processes on the self-regulatory variables.

Secondly, SPSS version 23.0 was used to estimate descriptive statistics (means and standard deviations) and the study variables’ bivariate correlations (Pearson correlation coefficient). Subsequently, the reliability of the subscales was analyzed using the Omega coefficient.

Thirdly, Mplus version 8 [39] was used to carry out Path Analysis with Robust Maximum Likelihood (MLR) estimation since it is robust to the non-normality of observations. The following fit indices were used to confirm an acceptable fit of the model to the data: × 2 value, Comparative Fit Index (CFI), Tucker-Lewis Index (TLI), Root Mean Square Error of Approximation (RMSEA), and Standardized Root Mean-square Residual (SRMR). Values greater than 0.90 and 0.95 for the CFI and TLI were considered to reflect “acceptable” and “excellent” model fit, respectively. Values smaller than 0.08 or 0.06 for the RMSEA and SRMR were considered to reflect “acceptable” and “excellent” model fit, respectively [40].

Fourthly, the path analysis was re-estimated using a maximum likelihood estimator and bootstrapping resampling procedures (*N* = 5000) to compute 95% bias-corrected confidence intervals (95% BcCI). The indirect association was deemed significant if the 95% BcCI did not include zero [41]. All models were statistically adjusted for sex and age.

Finally, for our secondary analysis, which hypothesized that the significant associations would differ in sub-groups with different weight change results, we used the same analytical procedures presented above. The sample was categorized according to the percentage of weight loss maintenance attained using known criteria [22, 42]. As a result, three groups were created: 1) Successfully lost weight – with weight losses above 3%; 2) Maintained weight – weight changed by less than 3%; and 3) Regained weight – with weight regain above 3%.

## Results

In brief, the NoHoW primary outcome findings showed that no changes in weight were observed (note that this was a weight loss maintenance intervention) and that there were no differences between the intervention arms in changes in weight or the mechanisms of action (see Supplemental file 1). Those findings are under review elsewhere.

Because no arm differences emerged, all the subsequent analysis was without considering the intervention arms. No significant changes (via paired comparisons, results not presented in a table – see Supplemental file) were observed from 0 to 12 months in weight, needs satisfaction, controlled forms of behavioral PA and eating regulations, and intrinsic goal contents. Small effect sizes (< 0.2) were observed for increases in autonomous motivation to exercise and eating, extrinsic goal contents, action plans, and coping plans. Action control decreased from baseline to 12 months (0. 358, medium effect size).

Table 1 presents the intercorrelations between the study’s variables. Increases in needs satisfaction, autonomous motivation, and self-regulatory processes/capacity were associated with better weight regain prevention. Conversely, an increase in controlled motivation was associated with an increase in weight. Virtual climate care, representing how participants perceived the intervention content, was positively associated with most putative mechanisms of action. Note that the strength of the associations is negligible in most variables, except for the self-regulatory processes/capacity, which tended to be medium.

**Table 1:** Intercorrelations between the logic model’s variables

		1		2		3		4		5		6		7		8		9		10		11	
1	Weight change 0–12 mo																						
2	Virtual Climate Care 6 mo	-.056																					
3	Basic Psy. Needs Global 0–6 mo	-.248	***	.126	***																		
4	Goal Content Intrinsic 0–6 mo	-.067	*	.152	***	.249	***																
5	Goal Content Extrinsic 0–6 mo	.017		-.012		.028		.449	***														
6	Reg. Eating Controled 0–6 mo	.086	**	-.032		-.124	***	.130	***	.242	***												
7	Reg. Eating Autonomous 0–6 mo	-.122	***	.106	**	.435	***	.296	***	.040		-.028											
8	Reg. Exercise Controled 0–6 mo	.127	***	-.033		-.104	***	.110	***	.247	***	.345	***	-.050									
9	Reg. Exercise Autonomous 0–6 mo	-.101	***	.075	*	.229	***	.297	***	.102	***	-.048		.280	***	.139	***						
10	Action Control 0–12 mo	-.406	***	.116	**	.178	***	.126	***	-.020		-.068	*	.130	***	-.047		.155	***				
11	Coping Plans 0–12 mo	-.311	***	.181	***	.258	***	.090	**	-.037		-.071	*	.150	***	-.077	*	.093	**	.375	***		
12	Action Plans 0–12 mo	-.343	***	.168	***	.265	***	.112	***	-.039		-.074	*	.165	***	-.079	*	.111	***	.437	***	.626	***

^*^p ≤ .05, **p ≤ .01, ***p ≤ .001; negative correlation with weight change means that the increase in the predictor was associated with 0–12 month weight reduction

Although we did not observe any overall mean weight changes, variability occurred when we looked at the individual level. Weight changes ranged from -26.3 to 30.6 kg, providing the necessary variability for the following set of analyses as we looked for mechanisms of action associated with different weight outcomes.

### Main analysis

**Fig. 2:**
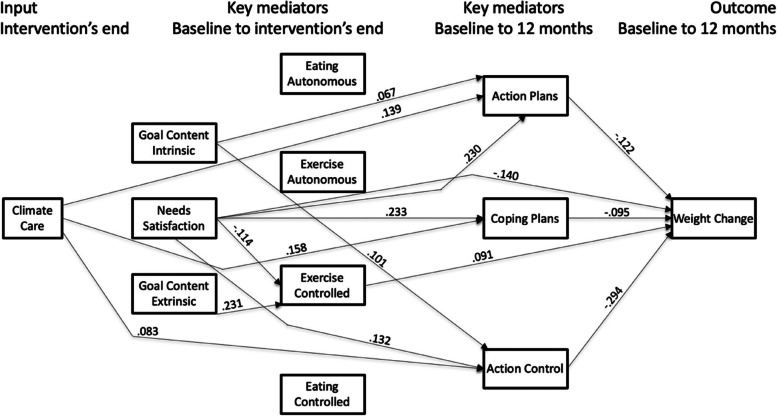
Path analysis: Estimates of the regression coefficients. Note: Only significant and direct paths are shown

The hypothesized model tested predicted 23.5% of weight change variance (*p* < 0.001), with an excellent fit: MLR χ2 = 23.351; df = 15; *p* = 0.126; CFI = 0.997; TLI = 0.985; RMSEA = 0.016 (90% CI = 0.000; 0.031); SRMR = 0.014. Figure 2 presents the direct associations between study variables.

Decreases in weight were predicted by improvements in needs satisfaction (β = -0.140), action plans (β  = -0.122), coping plans (β  = -0.095) and action control (β  = -0.294), and by increases in exercise controlled motivations (β  = 0.091). Note that due to how the variable’s changes were calculated, one should, for example, read that the negative value of the estimate for action control represents that improvements in action control were associated with more weight loss, a positive outcome.

An increase in action plans was predicted by improvements in climate care (β  = 0.139), goal content intrinsic (β  = 0.067), and needs satisfaction (β  = 0.230). Increases in coping plans were predicted by improvements in climate care (β  = 0.158), and needs satisfaction (β  = 0.233). An increase in action control was predicted by improvements in climate care (β  = 0.083), goal content intrinsic (β  = 0.101), and needs satisfaction (β  = 0.132). And finally, a decrease in exercise controlled motivation was predicted by improvements in psychological needs satisfaction (β  = -0.114), and by increases in goal content extrinsic (β  = 0.231).

For a complete table of the direct associations, please refer to the Supplementary file 2, Table 3.

Table 2 presents the indirect effects and shows that climate care was negatively and indirectly associated with weight change via action control (β  = -0.024, 95% BcCI = -0.045; -0.007), coping plans (β  = -0.015, 95% BcCI = -0.028; -0.006) and action plans (β  = -0.017, 95% BcCI = -0.031; -0.008). The results showed that higher scores of climate care improved weight change by increasing these three variables, representing a path by which participants better manage their weight loss (see also Table 3 in the discussion section for a summary of these results).

**Table 2:** Testing the logic model: standardized parameter estimates of indirect effects predicting weight change

**Variables**		
**Predictor**	Estimate	Bootstrap 95% CI
**Climate Care—> Weight Change 0–12 mo**		
Total Effect	-.032	-.084; .024
Direct Effect	.029	-.023; .082
Total Indirect	-.060	-.088; -.035
Indirect Effects Via		
Action Control 0–12 mo	-.024	-.045; -.007
Coping Plans 0–12 mo	-.015	-.028; -.006
Action Plans 0–12 mo	-.017	-.031; -.008
**Needs Satisfaction 0–6 mo—> Weight Change 0–12 mo**		
Total Effect	-.242	-.293; -.191
Direct Effect	-.140	-.191; -.089
Total Indirect	-.102	-.139; -.065
Indirect Effects Via		
Exercise Controlled Mot. 0-6mo	-.010	-.019; -.005
Action Control 0–12 mo	-.039	-.058; -.022
Coping Plans 0–12 mo	-.022	-.039; -.010
Action Plans 0–12 mo	-.028	-.045; -.014
**Goal Content Intrinsic 0–6 mo—> Weight Change 0–12 mo**		
Total Effect	-.017	-.070; .036
Direct Effect	.026	-.024; .077
Total Indirect	-.043	-.072; -.014
Indirect Effects Via		
Exercise Controlled Mot. 0–6 mo	.004	.000; .011
Action Control 0–12 mo	-.030	-.049; -.013
Action Plans 0–12 mo	-.008	-.019; -.002
**Goal Content Extrinsic 0–6 mo—> Weight Change 0–12 mo**		
Total Effect	.022	-.034; .081
Direct Effect	-.032	-.087; .022
Total Indirect	.055	.026; .085
Indirect Effects Via		
Exercise Controlled Mot. 0–6 mo	.021	.009; .035
Action Control 0–12 mo	.017	.001; .034
Coping Plans 0–12 mo	.006	.001; .016
Action Plans 0–12 mo	.009	.002; .020

Only significant indirect effects are shown. Negative coefficients represent more weight loss. Exercise Controlled Mot.—Exercise Controlled Motivation

**Table 3:** Summary of the significant paths in the primary analysis

Climate care -> Weight Change
More climate care -> more action control 0-12 mo -> weight loss 0-12 mo
More climate care -> more coping control 0-12 mo -> weight loss 0-12 mo
More climate care -> more action plans 0-12 mo -> weight loss 0-12 mo
Needs satisfaction -> Weight Change
More needs satisfaction 0-6 mo -> more exercise controlled motivation 0-6 mo -> weight loss 0-12 mo
More needs satisfaction 0-6 mo -> more action control 0-12 mo -> weight loss 0-12 mo
More needs satisfaction 0-6 mo -> more coping control 0-12 mo -> weight loss 0-12 mo
More needs satisfaction 0-6 mo -> more action plans 0-12 mo -> weight loss 0-12 mo
Goal contents intrinsic -> Weight Change
* More intrinsic goal contents 0*-*6 mo* -> *more exercise controlled motivation 0*-*6 mo* -> *weight regain* 0-12 mo
More intrinsic goal contents 0-6 mo -> more action control 0-12 mo -> weight loss 0-12 mo
More intrinsic goal contents 0-6 mo -> more action plans 0-12 mo -> weight loss 0-12 mo
Goal contents extrinsic -> Weight Change
* More extrinsic goal contents 0*-*6 mo* -> *more exercise controlled motivation 0*-*6 mo* -> *weight regain* 0–12 mo
* More extrinsic goal contents 0*-*6 mo* -> *more action control* 0-12 mo -> *weight regain* 0-12 mo
* More extrinsic goal contents 0*-*6 mo* -> *more coping plans* 0-12 mo -> *weight regain* 0-12 mo
* More extrinsic goal contents 0*-*6 mo* -> *more action plans* 0-12 mo -> *weight regain* 0-12 mo

The italics represent the weight regain indirect effect processes

Other results in Table 2 show that improvements in needs satisfaction led to better weight loss management via total, direct and indirect effects, representing a partial mediation process. The indirect effects showed that positive changes in needs satisfaction improved weight change outcomes via increases in action control (β = -0.039, 95% BcCI = -0.058; -0.022), coping plans (β = -0.022, 95% BcCI = -0.039; -0.010), action plans (β = -0.028, 95% BcCI = -0.045; -0.014), and exercise controlled motivation (β = -0.010, 95% BcCI = -0.019; -0.005). Improvements in action control, action plans, and coping plans were now joined by increases in exercise controlled motivation as paths for weight regain management.

Improvements in intrinsic goal contents were associated with better weight loss management. The indirect effects showed that positive changes in intrinsic goal contents improved weight change outcomes via increases in action control (β = -0.030, 95% BcCI = -0.049; -0.013), action plans (β = -0.008, 95% BcCI = -0.019; -0.002), and decreases in exercise controlled motivation (β = 0.004, 95% BcCI = 0.000; 0.011).

As expected, the results regarding the extrinsic goal contents were opposite to the previous results. Specifically, an increase in the extrinsic goal contents was associated with worse weight management outcomes via decreases in action control (β = 0.017, 95% BcCI = 0.001; 0.034), coping plans (β  = 0.006, 95% BcCI = 0.001; 0.016), action plans (β = 0.009, 95% BcCI = 0.002; 0.020), and exercise controlled motivation (β = 0.021, 95% BcCI = 0.009; 0.035).

Overall, higher scores of climate care and positive changes in needs satisfaction, intrinsic goal contents, action control, coping plans, action plans, and exercise controlled motivation represented paths for weight regain prevention. As predicted in the logic model, improvements in these variables positively affected weight regain prevention. However, although the logic model predicted that increases in exercise controlled motivation would lead to poorer weight regain management, the results showed that increases in this variable were also associated with better weight loss management.

In the initial analysis, we focused on a specific research question outlined in NoHoW's publication plan. However, we conducted additional analyses where the timeframes of variables did not overlap. For instance, motivational variables were examined within a timeframe of 0–6 months, while self-regulation variables were examined within a timeframe of 6–12 months. The results of these analyses showed that the model had low levels of data fit. This suggests that the expected temporal dissociation, as predicted by some theoretical models, was not observed in our trial. It is possible that some variables' changes may be intertwined rather than occurring as distinct, stage-like processes or that these psychosocial processes may represent independent paths.

### Secondary analysis

We observed a high level of variability in the weight regain prevention outcomes. We hypothesized that the psychological processes would predict weight change differently according to the participant’s body weight change. To categorize this variability, we created three groups following known criteria [22, 42]. The same statistical model was used, now looking at how the putative paths affected weight change separately in each group.

The hypothesized path model tested predicted 8.6% of the weight change variance in participants who successfully lost weight (*p* = 0.007), 9.0% in the ones who maintained (*p* = 0.001), and 17.6% in the ones who regained (*p* < 0.001), with excellent data fit: MLR χ2 = 56.28; df = 45; *p* = 0.121; CFI = 0.994; TLI = 0.967; RMSEA = 0.022 (90% CI = 0.000; 0.038); SRMR = 0.019 (see Supplementary file for the full results table and a summary of the results in Table 4).

**Table 4:** Summary of the group's path analysis

Successfully lost weight (> 3% weight loss 0-12 mo; *n* = 225)
More Intrinsic motives 0-6 mo -> more action control 0-12 mo -> weight loss 0-12 mo
Maintain weight (3% ≤ weight change 0-12 mo ≥ 3%; *n* = 349)
More Needs satisfaction 0-6 mo -> more autonomous eating motivation 0-6 mo -> weight loss 0-12 mo
More Intrinsic motives 0-6 mo -> more autonomous eating motivation 0-6 mo -> weight loss 0-12 mo
More Intrinsic motives 0-6 mo -> more controlled eating motivation 0-6 mo -> weight loss 0-12 mo
Regained weight (> 3% weight regain 0-12 mo; *n* = 296)
More Climate care -> more action plans 0-12 mo -> weight loss 0-12 mo
More Needs satisfaction 0-6 mo -> more action control 0-12 mo -> weight loss 0-12 mo
More Needs satisfaction 0-6 mo -> more action plans 0-12 mo -> weight loss 0-12 mo
* More Extrinsic motives* 0-6 mo -> *less action control 0*-*12 mo* -> *weight regain 0*-*12 mo*
* More Extrinsic motives* 0-6 mo -> *less controlled eating motivation* -> *weight regain 0*-*12 mo*
More Intrinsic motives 0-6 mo -> more action control 0-12 mo -> weight loss 0-12 mo

The italic represent the weight regain processes

Interestingly, in participants who successfully lost weight, only one putative path was significant, while in participants who maintained, the number of significant paths increased to three. Furthermore, in participants who regained, the number of significant paths was even higher (six). In this group, a total mediation was observed in the needs satisfaction model, in that increased needs satisfaction improved weight loss via increases in action plans and action control. It seems that the putative psychosocial processes played a lesser role when participants were able to lose weight. The role of the processes increased in the maintenance group and even more in the regain group, where they appear to be more critical in understanding the psychosocial processes involving weight regain.

## Discussion

This NoHoW-based study sought to determine if a path analytical model resulting from self-determination theory and self-regulation could explain body weight change for 12 months.

According to our main results, a quarter of the weight change variance (*r*2 = 23.5%) was achieved via the paths predicted in the logic model. In summary, our results highlight: a) the positive role of supportive climate care; b) that improvements in needs satisfaction and intrinsic goal contents lead to weight loss via improvements in self-regulatory skills; and c) that more extrinsic goals lead to weight regain via more exercise-related controlled motivation and more self-regulatory skills. While some of these results confirmed this study’s rationale, there was an exception: exercise controlled motivation played a mixed role in the associations’ dynamics between needs satisfaction, intrinsic and extrinsic goals, and weight changes.

Our findings extended previous studies with the Self-Determination Theory health behavior change model [23, 24] by adding self-regulatory processes as in-between mechanisms on the effect’s path from supportive climate care and needs satisfaction to intervention outcomes.

Focusing on the climate care results, although the bivariate correlations suggested several potential paths of influence in line with the logic model, the main analysis signaled that only the paths involving action plans, coping plans, and action control are significant. Looking at the logic model from right to left (i.e., outcomes to antecedents), one can hypothesize that these paths represent more robust mechanisms by sitting closer to the actual behaviors that lead to changes in weight. The self-regulatory variables also encompass the operationalization of eating and exercise autonomous regulations. More internalized regulations – the wantivation—are expected to provide the psychosocial stability to create and sustain adequate action plans and coping plans while feeling in control of one’s actions [5]. The needs satisfaction results support this hypothesis, as the bivariate correlations align with the logic model and the significant results from the mediation analysis focused on the paths involving action plans, coping plans, and action control.

Still concerning the supportive climate care data, a new, unexpected, path is suggested: exercise-controlled regulations represented a beneficial mechanism by which needs satisfaction influenced weight regain prevention efforts. We offer a similar explanation to the one presented above; self-regulatory processes are expected to benefit from a stable psychosocial context provided by internalized eating and exercise regulations. The unexpected exercise-controlled regulation results should be contextualized in the light of a new fact; participants used a FitBit tracker daily and averaged 10,600 steps at 12 months, attaining public health physical activity guidelines [43]. We hypothesize that a specific number of daily steps was part of the participant’s action plans. Alongside, coping plans may also focus on increasing the number of steps to compensate for a lapse in their dietary efforts, for example. These processes, which could be considered a sort of gamification with oneself (or even with others), fall into the controlled side of exercise regulations, albeit with less harmful results than the theory suggests. Assor et al. [44] has advocated disentangling the introjected regulation (a controlled form of motivation) considering its approach vs. avoidance components. Being better than before – the approach component of introjected regulation—does not necessarily represent a depleting form of regulation and can sustain behavior for long periods. We hypothesize that the steps-related action or coping plans fall into this approach component of introjected regulation (i.e., instead of in the avoidance approach, where one feels compelled to exercise to avoid guilt, for example). Future studies should look deeper into this hypothesis, searching for the potential deleterious effect of this strategy on psychosocial outcomes such as stress in avoidance-based vs. approach-based introjected regulations.

The goal content-related paths of the logic model presented an extra layer of information to this discussion. The intrinsic goal content model showed that increases in action plans and action control and decreases in exercise controlled-motivation predicted better weight regain prevention outcomes (i.e., wantivation). On the other hand, the extrinsic goal content model showed that increases in the significant indirect effects was associated with worse weight outcomes (i.e., mustivation). In this instance, we observed that increases in exercise-controlled motivation, alongside increases in action plans, coping plans, and action control led to increases in weight. The underlying mechanisms explaining these results may be that the introjected regulation's approach component is beneficial only when intrinsic goal content increases. In this scenario, action plans and action control align with the participant’s internalized motives. Conversely, when extrinsic motives increase, we expect that participants used external and avoidance-introjected regulations. This means that the action plans, coping plans, and action control would be instrumentalized by focusing on external and controlling outcomes (i.e., mustivation). This situation is not sustainable long-term, leading to worse weight prevention regain outcomes [5].

The secondary analysis (i.e., outcome-related groups) presented an interesting pattern. We observed increasing importance of the mechanisms of action from the group that lost weight (where only one path was significant) to the group that maintained weight (three significant paths) and to the regainers group (six significant paths). These findings suggest that when losing weight, the behavior change processes become automatic, habitual, and less conscious, and thus not captured via the self-reported questionnaires used in our measures of motivational and self-regulatory processes, which tap more conscious processes.

Maintaining weight seems to demand more conscious efforts, specifically while changing eating-related variables, which emerged as significant in the maintainer’s findings for the first time. As these processes are more conscious, the self-reported questionnaires captured these changes. Eating changes are commonly regarded as providing swifter changes in weight [45]; thus, it was expected that the NoHoW participants would react to their weight fluctuations by focusing on their eating. Interestingly, these efforts were only associated with weight loss when based on needs satisfaction or intrinsic goals antecedents (i.e., wantivation), confirming Self-Determination Theory’s predictions [2]. While not observed in this paper, we predict that changes in eating motivation may backfire and lead to further weight increases, especially if the goal content antecedent is extrinsic [5].

Finally, the regainers’ findings reflect the larger number of significant psychosocial processes as they struggle to manage their weight. Again, the results are aligned with the paper’s rationale: intrinsic nurturing antecedents lead to weight loss (i.e., wantivation), while extrinsic antecedents lead to weight regain (i.e., mustivation). The co-existence of significant weight loss and weight regain mechanisms in the participants who regained weight may reflect situations of weight cycling [46].

The success group findings present a unique opportunity to reflect and generate hypotheses on the underlying learning mechanisms leading to behavior maintenance. The rationale supporting these hypotheses can be summarized by: 1) the sentence “from conscious incompetence to sub-conscious competence” [47] is illustrative of the participant’s 12-month-long weight management experiences; and 2) the most internalized forms of self-regulation may represent how our consciousness (the rider, system 2) interacts with automaticity, habits, and sub-conscious processes (the elephant, system 1).

Accordingly, we suggest two hypotheses for future studies.

First, that automaticity or habit mechanisms – less conscious—are taking over the weight management behaviors of the most successful groups.This could explain the smaller number of significant, conscious, motivational, and self-regulatory mechanisms of action captured by the questionnaires in participants who successfuly lost or maintained weight (system 1 / the elephant relies on the newly learned habits). On the other hand, participants who regained weight present a higher number of significant conscious processes as they are still struggling to regulate their weight management behaviors (system 2 / the rider is looking for solutions). As a corollary, participants who regained weight are in the state of conscious incompetence, while successful participants are in the state of sub-conscious competence.

Second, that the internalization process of automaticity and habit formation is linked with autonomous motivational mechanisms in the sense that the autonomous motivational mechanisms provide energy to push and translate the conscious content to the sub-conscious. As such, the autonomous motivational mechanisms would be a path and a language that links system 2 / the rider to system 1 / the elephant. The studies on flow states, where “being in the zone” is linked to automaticity and habit processes, show that these states are linked to the highest levels of intrinsic motivation [48]. Hence, we suggest that wantivation facilitates new behaviors' learning and internalization processes leading to automaticity and habit formation.

### Limitations and future studies

Due to the absence of effects of the NoHoW intervention, the strength of the logic model pathways may have been diminished, thereby limiting the analytical capabilities of the study. In addition, we hypothesize that the dosage of the intervention, which consists of a digital-only behavioral change program comprising 17 digital sessions lasting approximately 5 to 10 min each over a span of 18 weeks, may not be sufficient to induce the expected motivational changes as outlined in the logic model. Therefore, it is important to acknowledge that this limitation hinders our analysis of the logic model and its associated theoretical rationale.

Furthermore, it is important to note that the full logic model of the NoHoW includes various emotional regulation variables that were not taken into account in this analysis. While the project has gathered data on emotional-related measures, we chose not to include them in the current analysis due to the overwhelming complexity it would have introduced. The inclusion of these variables would have resulted in a large number of interactions that were difficult to interpret, leading us to make the decision to analyze the emotional-regulation aspect separately in another paper.

The NoHoW study relied on questionnaires to measure psychosocial variables. The comprehensive logic model was translated into extensive measurement packages. Considering this extensiveness, we created two measurement sessions at each data collection point to reduce the participants' load. Even so, our process evaluation detected a few cases of participants stating that the psychometrics were demanding, albeit none declined to complete them. In addition, the internal consistency measures were good, signaling that the negative impact of extended measurements was limited.

The discrete data collection points—at baseline, six, and 12-months—may have limited the analysis of the dynamics between the factors in the NoHoW logic model. We recognize that the discrete data collection points at baseline, six, and 12 months may have restricted our analysis of the interplay between factors in the NoHoW logic model. Additionally, there are overlapping change variables in the model. For instance, the motivational variables from 0–6 months partially overlap with the self-regulation variables from 0–12 months. The decision to use these specific timeframes was informed by the best available evidence during the planning phase of this study. We aimed to capture the full extent of changes in putative predictors for self-regulation measures and how the intervention from 0–6 months impacted motivational variables, which would be associated and potentially influencing self-regulation variables (0–12 months). Alternative options were thoroughly discussed (e.g., 0–6 motivational affecting 6–12 self-regulation), but we ultimately executed the plan, decided about one year before the writing of this study.

The different sizes of the success groups, and measuring weight change within the subgroups may have led to a statistical artifact. The group who regained weight was larger, seconded by the maintenance, with the smaller group comprising the participants who successfully lost weight. However, this last group included 225 participants, a large enough sample to avoid Type I errors. A similar imbalance should be noted regarding the sex distribution. Still, the large sample included 293 men, and sex was included in the analytical models as a covariate, which limits this potential imbalance effect.

Automaticity and habit – two variables mentioned in our hypothesis generation section—represented a confounding variable in our study, as we did not measure them. We suggest that future studies investigate this – measuring habits, motives, self-regulation, resources, and environmental and social influences, as people learn to maintain a behavior. We predict that such studies may show an increase in habits and a decrease in motives and self-regulatory processes in the successful participants.

Conversely, this article's strengths lie in its large sample size and the analytical procedure. Data was gathered over 12 months from a controlled trial in three European countries, with validated and standardized methods used in all centers. While replication is necessary, the results may apply to several European regions, given the large samples collected in the three countries representing Scandinavia, the UK, and Southern Europe. The mediation analysis examines the mechanisms of action of the NoHoW – a complex intervention, providing results to aid future programs in preventing weight regain.

## Conclusion

In conclusion, our results showed that the logic model’s key paths were significant in a digital behavior change intervention aiming at long-term weight regain prevention. The most effective path involved need satisfaction and intrinsic motives leading to improved self-regulatory skills and better weight change outcomes – a wantivation process. On the other hand, extrinsic motives lead to more self-regulatory skills linked to worse weight regain prevention, signaling that mustivation processes will provide energy but not a good direction for our behavioral efforts. These findings extend both the Self-Determination Theory and Self-Regulation rationales in that they link motivational and self-regulatory skills processes.

In addition, the study generated hypotheses on how motivation-related (i.e., conscious) variables may be linked with habits and other sub-conscious processes; behavior internalization is hypothesized to be a function of autonomous motivation feeding habit formation. We are certainly looking to (or to see others) study this hypothesis in the future.

### Supplementary Information

**Additional file 1. **Tables supplementing the main analysis presented in the manuscript.

## Data Availability

There are legal restrictions on sharing data that contain potentially identifying or sensitive person information. The restrictions are imposed by The Danish Data Protection Agency (https://www.datatilsynet.dk/english/). The data is being held officially by the James Hutton Institute and are available upon request. Contact details for the James Hutton Institute are as follows: The James Hutton Institute |Craigiebuc kler|Aberdeen AB15 8QH|Scotland UK|Tel: + 44(0)344 9285428 or alternatively visit the EASO NoHoW website at https://easo.org/thenohow-dataset/.

## Abbreviations

SDT: Self-Determination Theory
BCT: Behavior change techniques
DBCI: Digital-based behavior change interventions
VCCQ: Virtual Care Climate Questionnaire
BPNSS: Basic Psychological Needs Satisfaction Scale
GCWMS: Goal Content for Weight Management Scale
REBS: Regulations for Eating Behaviour Scale
BREQ-3: Behavioral Regulations for Exercise Questionnaire
CFI: Comparative Fit Index
TLI: Tucker-Lewis Index
RMSEA: Root Mean Square Error of Approximation
SRMR: Standardized Root Mean-square Residual
BcCI: 95% Bias-corrected confidence intervals
MLR: Robust Maximum Likelihood
