# Supplementary material for: Are motivational and self-regulation factors associated with 12 months’ weight regain prevention in the NoHoW study? An analysis of European adults
Source: Int J Behav Nutr Phys Act. 2023 Oct 27;20:128. doi: 10.1186/s12966-023-01529-8 (PMC10605649; doi:10.1186/s12966-023-01529-8)
Supplement: Supplementary file 1 — Additional file 1. Tables supplementing the main analysis presented in the manuscript. [file 12966_2023_1529_MOESM1_ESM.pdf]

Supplemental File

Table of contents

RELIABILITY SCORES 1

PAIRED COMPARISONS FOR THE VARIABLES INCLUDED IN THE STUDY 2

INTERVENTION ARM EFFECTS ON THE THEORETICAL MECHANISMS OF ACTION 3

COMPLETE RESULTS OF THE DIRECT ASSOCIATIONS (LINKED WITH FIGURE ESTIMATES OF THE REGRESSION COEFFICIENTS) 20

STANDARDIZED PARAMETER ESTIMATES OF INDIRECT EFFECTS BY SUCCESS IN WEIGHT REGAIN PREVENTION GROUPS 24

Reliability scores

Table 1 – Reliability scores (McDonalds' Omega)

(Sub)Scale	Baseline	6-month	12-month
Needs Satisfaction	.916	.934	.946
Goal Content Ext.	.868	.807	.825
Goal Content Intr.	.886	.904	.901
Exercise Amotivation	.807	.824	.828
Exercise External	.861	.857	.873
Exercise Introjected	.793	.784	.811

Exercise Identified	.825	.830	.820
Exercise Integrated	.915	.921	.922
Exercise Intrinsic	.919	.919	.927
Eating Amotivation	.821	.802	.783
Eating External	.847	.868	.877
Eating Introjected	.734	.742	.773
Eating Identified	.838	.866	.860
Eating Integrated	.844	.869	.880
Eating Intrinsic	.895	.917	.914
Action Planning	.810	.858	.876
Coping Planning	.945	.953	.959
Action Control Scale	.895	.894	.904

Paired comparisons for the variables included in the study

Table 2 – Paired comparisons for the variables included in the study
(note that a negative mean difference represents an increase from the initial to the measures)

Pairs	Variables	Mean	Std. Deviation	95% CI of the Difference		t	Sig. (2-tailed)
Pair 1	Weight	.09920	6.02334	-.30161	.50000	.486	.627
Pair 2	Basic Psychological Needs	.05779	.94599	-.00750	.12307	1.737	.083
Pair 3	Goal Content Challenge	-.03417	1.27680	-.12213	.05378	-.763	.446
Pair 4	Goal Content Social	-.13055	1.20463	-.21338	-.04773	-3.094	.002
Pair 5	Goal Content Image	-.08477	1.04970	-.15698	-.01255	-2.304	.021
Pair 6	Goal Content Health	-.00398	.86464	-.06340	.05543	-.132	.895
Pair 7	Exercise Controlled Motivation	.01009	.90449	-.05199	.07216	.319	.750

Pair 8	Exercise Autonomous Motivation	-.16227	.87563	-.22248	-.10206	-5.290	.000
Pair 9	Eating Controlled Motivation	-.00656	1.03349	-.07771	.06459	-.181	.856
Pair 10	Eating Autonomous Motivation	-.11808	.96751	-.18469	-.05148	-3.480	.001
Pair 11	Action Control	.28014	.98466	.21128	.34899	7.986	.000
Pair 12	Coping Plans	-.13740	.86477	-.19799	-.07681	-4.452	.000
Pair 13	Action Plans	-.12803	.87163	-.18909	-.06696	-4.115	.000

Intervention arm effects on the theoretical mechanisms of action

No group differences were observed in the variables considered in this study.

The study’s logic model is presented below for easier variables’ reference.

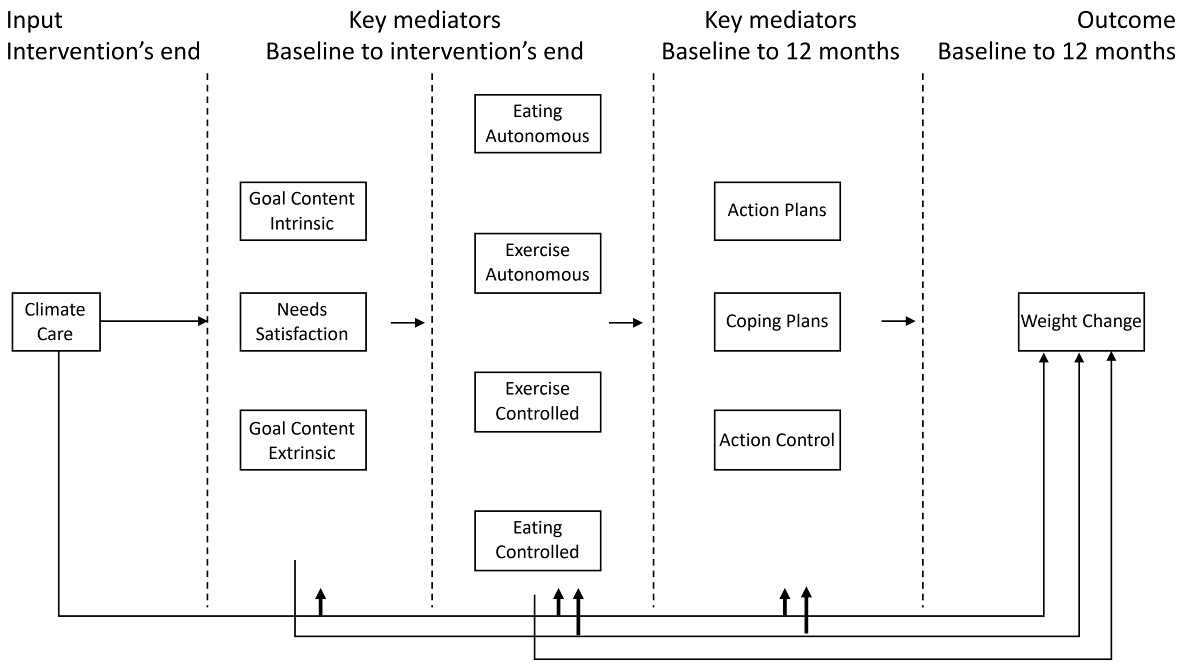

Climate Care

ANOVA - VCCTt

Cases	Sum of Squares	df	Mean Square	F	p	η²
study_arm_id	3.589	2	1.795	1.853	0.158	0.006
Residuals	646.825	668	0.968			

Note. Type III Sum of Squares

Descriptives - VCCTt

study_arm_id	N	Mean	SD	SE	Coefficient of Variation
2	215	4.740	0.987	0.067	0.208
3	229	4.652	0.987	0.065	0.212

Descriptives - VCCTt

study_arm_id	N	Mean	SD	SE	Coefficient of Variation
4	227	4.829	0.978	0.065	0.203

(only the intervention arms are included in this analysis)

Goal Content Intrinsic

Within Subjects Effects

Cases	Sum of Squares	df	Mean Square	F	p	η^2_p
Goal Content Chalenge	0.148	1	0.148	0.177	0.674	1.394×10^{-4}
Goal Content Chalenge ✻ study_arm_id	1.682	3	0.561	0.671	0.570	0.002
Goal Content Chalenge ✻ age	2.736	1	2.736	3.272	0.071	0.003
Goal Content Chalenge ✻ gen	0.550	1	0.550	0.658	0.417	5.179×10^{-4}
Residuals	1061.736	1270	0.836			

Note. Type III Sum of Squares

Between Subjects Effects

Cases	Sum of Squares	df	Mean Square	F	p	η^2_p
study_arm_id	7.125	3	2.375	0.789	0.500	0.002
age	1.181	1	1.181	0.393	0.531	3.091×10^{-4}
gen	15.704	1	15.704	5.220	0.022	0.004
Residuals	3820.500	1270	3.008			

Note. Type III Sum of Squares

Descriptives

Goal Content Chalenge	study_arm_id	N	Mean	SD	SE	Coefficient of Variation

Within Subjects Effects

Cases		Sum of Squares	df	Mean Square	F	p	η^2_p
Baseline	1	313 4.575	1.410	0.080		0.308	
	2	312 4.531	1.386	0.078		0.306	
	3	333 4.622	1.428	0.078		0.309	
	4	318 4.516	1.416	0.079		0.314	
6months	1	313 4.625	1.342	0.076		0.290	
	2	312 4.661	1.336	0.076		0.287	
	3	333 4.700	1.387	0.076		0.295	
	4	318 4.504	1.395	0.078		0.310	

Within Subjects Effects

Cases	Sum of Squares	df	Mean Square	F	p	η^2_p
Goal Content health	1.276	1	1.276	3.330	0.068	0.003
Goal Content health ✻ study_arm_id	0.026	3	0.009	0.023	0.995	5.326×10^{-5}
Goal Content health ✻ age	0.104	1	0.104	0.271	0.603	2.123×10^{-4}
Goal Content health ✻ gen	3.417	1	3.417	8.919	0.003	0.007
Residuals	488.128	1274	0.383			

Note. Type III Sum of Squares

Between Subjects Effects

Cases	Sum of Squares	df	Mean Square	F	p	η^2_p
study_arm_id	1.813	3	0.604	0.431	0.731	0.001
age	29.844	1	29.844	21.304	< .001	0.016
gen	0.241	1	0.241	0.172	0.678	1.353×10^{-4}
Residuals	1784.670	1274	1.401			

Between Subjects Effects

Cases	Sum of Squares	df	Mean Square	F	p	η^2_p

Note. Type III Sum of Squares

Descriptives

Descriptives

Goal Content health	study_arm_id	N	Mean	SD	SE	Coefficient of Variation
Baseline	1	314	5.971	0.917	0.052	0.154
	2	313	5.928	1.033	0.058	0.174
	3	335	5.945	0.991	0.054	0.167
	4	318	5.997	0.929	0.052	0.155
6months	1	314	5.977	0.923	0.052	0.154
	2	313	5.915	1.009	0.057	0.171
	3	335	5.943	0.883	0.048	0.149
	4	318	5.994	0.912	0.051	0.152

Goal Content Extrinsic

Within Subjects Effects

Cases	Sum of Squares	df	Mean Square	F	p	η^2_p
Goal Content Image	1.466	1	1.466	2.472	0.116	0.002
Goal Content Image ✻ study_arm_id	2.394	3	0.798	1.346	0.258	0.003
Goal Content Image ✻ age	0.339	1	0.339	0.571	0.450	4.484×10^{-4}
Goal Content Image ✻ gen	0.302	1	0.302	0.509	0.476	3.999×10^{-4}
Residuals	755.042	1273	0.593			

Note. Type III Sum of Squares

Between Subjects Effects

Cases	Sum of Squares	df	Mean Square	F	p	$η^2_p$
study_arm_id	3.018	3	1.006	0.433	0.729	0.001
age	103.485	1	103.485	44.579	< .001	0.034
gen	43.149	1	43.149	18.587	< .001	0.014
Residuals	2955.161	1273	2.321			

Note. Type III Sum of Squares

Descriptives

Goal Content Image	study_arm_id	N	Mean	SD	SE	Coefficient of Variation
Baseline	1	313	5.282	1.238	0.070	0.234
	2	314	5.354	1.206	0.068	0.225
	3	334	5.290	1.305	0.071	0.247
	4	318	5.302	1.245	0.070	0.235
6months	1	313	5.411	1.211	0.068	0.224
	2	314	5.426	1.203	0.068	0.222
	3	334	5.418	1.198	0.066	0.221
	4	318	5.278	1.209	0.068	0.229

Within Subjects Effects

Cases	Sum of Squares	df	Mean Square	F	p	$η^2_p$
Goal Content Social	0.108	1	0.108	0.149	0.700	$1.167×10^{-4}$
Goal Content Social ✻ study_arm_id	4.579	3	1.526	2.096	0.099	0.005
Goal Content Social ✻ age	0.057	1	0.057	0.079	0.779	$6.175×10^{-5}$
Goal Content Social ✻ gen	3.402	1	3.402	4.672	0.031	0.004
Residuals	927.762	1274	0.728			

Note. Type III Sum of Squares

Between Subjects Effects

Cases	Sum of Squares	df	Mean Square	F	p	η^2_p
study_arm_id	21.406	3	7.135	1.766	0.152	0.004
age	49.446	1	49.446	12.237	< .001	0.010
gen	20.818	1	20.818	5.152	0.023	0.004
Residuals	5148.040	1274	4.041			

Note. Type III Sum of Squares

Descriptives

Goal Content Social	study_arm_id	N	Mean	SD	SE	Coefficient of Variation
Baseline	1	314	3.711	1.537	0.087	0.414
	2	314	3.706	1.476	0.083	0.398
	3	334	3.799	1.518	0.083	0.400
	4	318	3.623	1.539	0.086	0.425
6months	1	314	3.924	1.595	0.090	0.406
	2	314	3.959	1.569	0.089	0.396
	3	334	3.980	1.631	0.089	0.410
	4	318	3.657	1.538	0.086	0.421

Basic Psychological Needs

Within Subjects Effects

Cases	Sum of Squares	df	Mean Square	F	p	η^2_p
Basic Psychological Needs	2.240	1	2.240	4.281	0.039	0.003
Basic Psychological Needs ✻ study_arm_id	3.548	3	1.183	2.260	0.080	0.005
Basic Psychological Needs ✻ age	6.455	1	6.455	12.335	< .001	0.010

Within Subjects Effects

Cases	Sum of Squares	df	Mean Square	F	p	η^2_p
Basic Psychological Needs ✻ gen	1.389	1	1.389	2.653	0.104	0.002
Residuals	662.516	1266	0.523			

Note. Type III Sum of Squares

Between Subjects Effects

Cases	Sum of Squares	df	Mean Square	F	p	η^2_p
study_arm_id	0.372	3	0.124	0.085	0.968	2.012×10^{-4}
age	1.579	1	1.579	1.082	0.298	8.543×10^{-4}
gen	19.157	1	19.157	13.130	< .001	0.010
Residuals	1847.169	1266	1.459			

Note. Type III Sum of Squares

Descriptives

Basic Psychological Needs	study_arm_id	N	Mean	SD	SE	Coefficient of Variation
Baseline	1	316	5.580	0.909	0.051	0.163
	2	311	5.644	0.879	0.050	0.156
	3	331	5.567	0.938	0.052	0.168
	4	314	5.515	0.963	0.054	0.175
6months	1	316	5.516	1.071	0.060	0.194
	2	311	5.432	1.116	0.063	0.206
	3	331	5.470	1.029	0.057	0.188
	4	314	5.512	1.071	0.060	0.194

Exercise motivation

Within Subjects Effects

Cases	Sum of Squares	df	Mean Square	F	p	η^2_p
Exercise Autonomous Motivation	0.813	1	0.813	2.075	0.150	0.002
Exercise Autonomous Motivation ✻ study_arm_id	2.218	3	0.739	1.888	0.130	0.004
Exercise Autonomous Motivation ✻ age	3.684	1	3.684	9.405	0.002	0.007
Exercise Autonomous Motivation ✻ gen	0.068	1	0.068	0.174	0.677	1.365×10^{-4}
Residuals	498.689	1273	0.392			

Note. Type III Sum of Squares

Between Subjects Effects

Cases	Sum of Squares	df	Mean Square	F	p	η^2_p
study_arm_id	0.394	3	0.131	0.040	0.989	9.533×10^{-5}
age	24.779	1	24.779	7.633	0.006	0.006
gen	114.665	1	114.665	35.323	< .001	0.027
Residuals	4132.390	1273	3.246			

Note. Type III Sum of Squares

Descriptives

Exercise Autonomous Motivation	study_arm_id	N	Mean	SD	SE	Coefficient of Variation
Baseline	1	315	4.956	1.342	0.076	0.271
	2	313	4.892	1.339	0.076	0.274
	3	335	4.888	1.471	0.080	0.301
	4	316	4.879	1.379	0.078	0.283
6months	1	315	5.002	1.393	0.078	0.278
	2	313	5.053	1.270	0.072	0.251
	3	335	5.094	1.411	0.077	0.277
	4	316	5.045	1.339	0.075	0.265

Within Subjects Effects

Cases	Sum of Squares	df	Mean Square	F	p	η^2_p
Exercise Controlled Motivation	0.015	1	0.015	0.038	0.846	2.957×10^{-5}
Exercise Controlled Motivation ✻ study_arm_id	0.192	3	0.064	0.157	0.925	3.689×10^{-4}
Exercise Controlled Motivation ✻ age	0.338	1	0.338	0.830	0.362	6.502×10^{-4}
Exercise Controlled Motivation ✻ gen	0.247	1	0.247	0.607	0.436	4.754×10^{-4}
Residuals	519.077	1276	0.407			

Note. Type III Sum of Squares

Between Subjects Effects

Cases	Sum of Squares	df	Mean Square	F	p	η^2_p
study_arm_id	4.412	3	1.471	0.967	0.407	0.002
age	14.551	1	14.551	9.573	0.002	0.007
gen	0.471	1	0.471	0.310	0.578	2.428×10^{-4}
Residuals	1939.575	1276	1.520			

Note. Type III Sum of Squares

Descriptives

Exercise Controlled Motivation	study_arm_id	N	Mean	SD	SE	Coefficient of Variation
Baseline	1	315	2.849	1.039	0.059	0.365
	2	314	2.917	0.968	0.055	0.332
	3	336	2.898	0.991	0.054	0.342
	4	317	2.794	0.920	0.052	0.329
6months	1	315	2.852	0.979	0.055	0.343
	2	314	2.904	0.997	0.056	0.343
	3	336	2.924	1.013	0.055	0.346
	4	317	2.824	0.959	0.054	0.340

Eating motivation

Within Subjects Effects

Cases	Sum of Squares	df	Mean Square	F	p	η^2_p
Eating Autonomous Motivation	1.125	1	1.125	2.175	0.141	0.002
Eating Autonomous Motivation ✻ study_arm_id	1.266	3	0.422	0.816	0.485	0.002
Eating Autonomous Motivation ✻ age	0.868	1	0.868	1.677	0.196	0.001
Eating Autonomous Motivation ✻ gen	3.302	1	3.302	6.384	0.012	0.005
Residuals	660.102	1276	0.517			

Note. Type III Sum of Squares

Between Subjects Effects

Cases	Sum of Squares	df	Mean Square	F	p	η^2_p
study_arm_id	0.305	3	0.102	0.053	0.984	1.238×10^{-4}
age	17.094	1	17.094	8.854	0.003	0.007
gen	37.366	1	37.366	19.356	< .001	0.015
Residuals	2463.353	1276	1.931			

Note. Type III Sum of Squares

Descriptives

Eating Autonomous Motivation	study_arm_id	N	Mean	SD	SE	Coefficient of Variation
Baseline	1	317	5.232	1.013	0.057	0.194
	2	313	5.196	1.101	0.062	0.212
	3	336	5.196	1.166	0.064	0.224
	4	316	5.204	1.141	0.064	0.219
6months	1	317	5.249	1.106	0.062	0.211

Descriptives

Eating Autonomous Motivation	study_arm_id	N	Mean	SD	SE	Coefficient of Variation
	2	313	5.319	1.140	0.064	0.214
	3	336	5.322	1.094	0.060	0.206
	4	316	5.279	1.164	0.065	0.220

Within Subjects Effects

Cases	Sum of Squares	df	Mean Square	F	p	η^2_p
Eating Controled Motivation	1.931	1	1.931	3.752	0.053	0.003
Eating Controled Motivation ✻ study_arm_id	3.923	3	1.308	2.541	0.055	0.006
Eating Controled Motivation ✻ age	1.968	1	1.968	3.824	0.051	0.003
Eating Controled Motivation ✻ gen	0.045	1	0.045	0.088	0.766	6.915×10^{-5}
Residuals	657.689	1278	0.515			

Note. Type III Sum of Squares

Between Subjects Effects

Cases	Sum of Squares	df	Mean Square	F	p	η^2_p
study_arm_id	9.248	3	3.083	1.637	0.179	0.004
age	2.969	1	2.969	1.577	0.209	0.001
gen	0.129	1	0.129	0.069	0.793	5.367×10^{-5}
Residuals	2406.353	1278	1.883			

Note. Type III Sum of Squares

Descriptives

Eating Controled Motivation	study_arm_id	N	Mean	SD	SE	Coefficient of Variation
Baseline	1	317	3.133	1.099	0.062	0.351
	2	314	3.216	1.159	0.065	0.361

Descriptives

Eating Controled Motivation	study_arm_id	N	Mean	SD	SE	Coefficient of Variation
	3	336	3.227	1.066	0.058	0.330
	4	317	3.198	1.075	0.060	0.336
6months	1	317	3.177	1.076	0.060	0.339
	2	314	3.260	1.154	0.065	0.354
	3	336	3.371	1.105	0.060	0.328
	4	317	3.120	1.021	0.057	0.327

Action Plans

Within Subjects Effects

Cases	Sum of Squares	df	Mean Square	F	p	η^2_p
Action Plan	0.565	1	0.565	1.373	0.242	0.001
Action Plan ✻ study_arm_id	0.467	3	0.156	0.378	0.769	9.965×10^{-4}
Action Plan ✻ age	2.400	1	2.400	5.829	0.016	0.005
Action Plan ✻ gen	0.004	1	0.004	0.009	0.924	8.019×10^{-6}
Residuals	468.124	1137	0.412			

Note. Type III Sum of Squares

Between Subjects Effects

Cases	Sum of Squares	df	Mean Square	F	p	η^2_p
study_arm_id	1.524	3	0.508	0.562	0.640	0.001
age	10.940	1	10.940	12.103	< .001	0.011
gen	5.000	1	5.000	5.531	0.019	0.005
Residuals	1027.700	1137	0.904			

Note. Type III Sum of Squares

Descriptives

Action Plan	study_arm_id	N	Mean	SD	SE	Coefficient of Variation
Baseline	1	290	3.546	0.779	0.046	0.220
	2	278	3.541	0.821	0.049	0.232
	3	287	3.537	0.712	0.042	0.201
	4	288	3.613	0.759	0.045	0.210
12months	1	290	3.634	0.909	0.053	0.250
	2	278	3.598	0.906	0.054	0.252
	3	287	3.661	0.814	0.048	0.222
	4	288	3.673	0.801	0.047	0.218

Coping Plans

Within Subjects Effects

Cases	Sum of Squares	df	Mean Square	F	p	η^2_p
Coping Plans	0.110	1	0.110	0.287	0.592	2.523×10^{-4}
Coping Plans ✻ study_arm_id	0.845	3	0.282	0.734	0.532	0.002
Coping Plans ✻ age	0.420	1	0.420	1.095	0.296	9.628×10^{-4}
Coping Plans ✻ gen	0.230	1	0.230	0.598	0.439	5.266×10^{-4}
Residuals	435.711	1136	0.384			

Note. Type III Sum of Squares

Between Subjects Effects

Cases	Sum of Squares	df	Mean Square	F	p	η^2_p
study_arm_id	1.260	3	0.420	0.451	0.717	0.001
age	6.446	1	6.446	6.917	0.009	0.006
gen	0.322	1	0.322	0.345	0.557	3.038×10^{-4}

Between Subjects Effects

Cases	Sum of Squares	df	Mean Square	F	p	η^2_p
Residuals	1058.647	1136	0.932			

Note. Type III Sum of Squares

Descriptives

Coping Plans	study_arm_id	N	Mean	SD	SE	Coefficient of Variation
Baseline	1	289	3.092	0.786	0.046	0.254
	2	279	3.047	0.755	0.045	0.248
	3	288	3.035	0.741	0.044	0.244
	4	286	3.064	0.775	0.046	0.253
12months	1	289	3.164	0.891	0.052	0.282
	2	279	3.107	0.874	0.052	0.281
	3	288	3.162	0.828	0.049	0.262
	4	286	3.217	0.836	0.049	0.260

Action Control

Within Subjects Effects

Cases	Sum of Squares	df	Mean Square	F	p	η^2_p
Action Control	1.257	1	1.257	2.495	0.114	0.002
Action Control ✻ study_arm_id	2.145	3	0.715	1.420	0.235	0.003
Action Control ✻ age	9.045	1	9.045	17.959	< .001	0.014
Action Control ✻ gen	8.678	1	8.678	17.230	< .001	0.013
Residuals	638.657	1268	0.504			

Note. Type III Sum of Squares

Between Subjects Effects

Cases	Sum of Squares	df	Mean Square	F	p	η^2_p
study_arm_id	0.528	3	0.176	0.171	0.916	4.035×10^{-4}
age	39.479	1	39.479	38.265	< .001	0.029
gen	11.823	1	11.823	11.460	< .001	0.009
Residuals	1308.231	1268	1.032			

Note. Type III Sum of Squares

Descriptives

Action Control	study_arm_id	N	Mean	SD	SE	Coefficient of Variation
Baseline	1	315	3.574	0.859	0.048	0.240
	2	312	3.655	0.869	0.049	0.238
	3	331	3.634	0.883	0.049	0.243
	4	316	3.627	0.879	0.049	0.242
12months	1	315	3.418	0.911	0.051	0.267
	2	312	3.359	0.897	0.051	0.267
	3	331	3.408	0.923	0.051	0.271
	4	316	3.468	0.910	0.051	0.262

Weight (please note that these results are under review in the main study paper, so the results should not be disseminated)

Within Subjects Effects

Cases	Sum of Squares	df	Mean Square	F	p	η^2_p
Weight	32.725	1	32.725	1.776	0.183	0.001
Weight ✻ study_arm_id	15.192	3	5.064	0.275	0.844	6.509×10^{-4}
Weight ✻ age	187.761	1	187.761	10.190	0.001	0.008
Weight ✻ gen	38.803	1	38.803	2.106	0.147	0.002

Within Subjects Effects

Cases	Sum of Squares	df	Mean Square	F	p	η^2_p
Residuals	23326.357	1266	18.425			

Note. Type III Sum of Squares

Between Subjects Effects

Cases	Sum of Squares	df	Mean Square	F	p	η^2_p
study_arm_id	271.935	3	90.645	0.183	0.908	4.324×10^{-4}
age	1309.118	1	1309.118	2.636	0.105	0.002
gen	65791.558	1	65791.558	132.492	< .001	0.095
Residuals	628659.462	1266	496.571			

Note. Type III Sum of Squares

Descriptives

Weight	study_arm_id	N	Mean	SD	SE	Coefficient of Variation
Baseline	1	320	84.226	17.312	0.968	0.206
	2	314	84.341	16.157	0.912	0.192
	3	323	83.317	16.738	0.931	0.201
	4	315	84.501	16.171	0.911	0.191
12months	1	320	84.269	18.162	1.015	0.216
	2	314	84.223	16.357	0.923	0.194
	3	323	83.486	16.796	0.935	0.201
	4	315	84.232	16.794	0.946	0.199

Complete results of the direct associations (linked with Figure Estimates of the regression coefficients)

Table 3 - Complete results of the direct associations (linked with Figure Estimates of the regression coefficients)

	Estimate	S.E.	Est./S.E.	Two-Tailed P-Value
WC012 ON				
VCCTT	0.029	0.032	0.896	0.370
BPNSG06	-0.140	0.031	-4.529	0.000
GCI06M	0.026	0.031	0.841	0.400
GCE06M	-0.032	0.033	-0.972	0.331
BREQA06	-0.033	0.031	-1.090	0.276
BREQC06	0.091	0.032	2.882	0.004
REBA06	0.011	0.031	0.353	0.724
REBC06	0.012	0.031	0.393	0.694
ACTIONC012	-0.294	0.029	-10.276	0.000
CPLAN012	-0.095	0.034	-2.800	0.005
APLAN012	-0.122	0.036	-3.360	0.001
GEN	-0.033	0.026	-1.284	0.199
AGE	-0.004	0.026	-0.160	0.873
BREQA06 ON				
VCCTT	0.027	0.035	0.783	0.434
BPNSG06	0.160	0.031	5.111	0.000
GCI06M	0.261	0.031	8.316	0.000
GCE06M	-0.013	0.033	-0.393	0.694
GEN	-0.029	0.025	-1.177	0.239
AGE	0.027	0.029	0.919	0.358

BREQC06 ON				
VCCTT	-0.033	0.036	-0.904	0.366
BPNSG06	-0.114	0.030	-3.796	0.000
GCI06M	0.044	0.032	1.366	0.172
GCE06M	0.231	0.031	7.381	0.000
GEN	-0.016	0.028	-0.583	0.560
AGE	-0.036	0.028	-1.267	0.205
REBA06 ON				
VCCTT	0.040	0.035	1.149	0.250
BPNSG06	0.378	0.036	10.565	0.000
GCI06M	0.227	0.030	7.474	0.000
GCE06M	-0.069	0.029	-2.429	0.015
GEN	0.036	0.026	1.405	0.160
AGE	0.018	0.025	0.727	0.467
REBC06 ON				
VCCTT	-0.029	0.034	-0.840	0.401
BPNSG06	-0.141	0.033	-4.330	0.000
GCI06M	0.078	0.032	2.447	0.014
GCE06M	0.211	0.031	6.740	0.000
GEN	-0.038	0.027	-1.392	0.164
AGE	-0.042	0.029	-1.434	0.151
ACTIONC0 ON				
VCCTT	0.083	0.037	2.262	0.024
BPNSG06	0.132	0.035	3.761	0.000
GCI06M	0.101	0.036	2.830	0.005

GCE06M	-0.057	0.034	-1.665	0.096
GEN	-0.057	0.028	-2.007	0.045
AGE	0.119	0.028	4.201	0.000
CPLAN012 ON				
VCCTT	0.158	0.037	4.248	0.000
BPNSG06	0.233	0.036	6.450	0.000
GCI06M	0.039	0.037	1.053	0.292
GCE06M	-0.062	0.038	-1.631	0.103
GEN	0.035	0.029	1.178	0.239
AGE	0.035	0.028	1.234	0.217
APLAN012 ON				
VCCTT	0.139	0.036	3.835	0.000
BPNSG06	0.230	0.037	6.208	0.000
GCI06M	0.067	0.034	1.958	0.050
GCE06M	-0.072	0.037	-1.953	0.051
GEN	0.012	0.027	0.439	0.660
AGE	0.079	0.029	2.767	0.006

**Variable in the table**	**Variable name**
ACTIONC012	Action Control, 0-12 mo
age	Age
Aplan012	Action Plans, 0-12 mo
BPNSG06	Satisfaction of the Basic Psychological Needs, 0-6 mo

BREQA06	Autonomous Exercise Regulation, 0-6 mo
BREQC06	Controlled Exercise Regulation, 0-6 mo
Cplan012	Coping Plans, 0-12 mo
GCE06M	Extrinsic Goal Content, 0-6 mo
GCI06M	Intrinsic Goal Content, 0-6 mo
gen	Gender
REBA06	Autonomous Eating Regulation, 0-6 mo
REBC06	Controlled Eating Regulation, 0-6 mo
VCCTt	Virtual Climate Care at the end of the intervention
WC012	Weight change, 0-12 mo

Standardized parameter estimates of indirect effects by success in weight regain prevention groups

Model	Model Fit		Variables		
	Chi-square/df	56.28/45; p=,121	<u>**Successful Losers**</u>		
Outcome Multigroup	RMSEA	0,022	**Predictor**	Estimate	Bootstrap 95% CI
Weight change 0-12	90% CI	0.000 to 0.038	Climate Care -> Weight Change		
	CFI	0,994	Total Effect	0.011	-0.104; 0.128
	SRMR	0,019	Direct Effect	0,028	-0.090; 0.131
	TLI	0,967	Total Indirect	-0,017	-0.058; 0.025
	Chi-Square Contributions		**Indirect Effects Via**		
	Successful Losers	16.27	None		
	Maintainers	16.91	**Predictor**	Estimate	Bootstrap 95% CI
	Regainers	23.09	Satisfaction Basic Psychological Needs -> Weight Change		
			Total Effect	-0,122	-0.232; -0.031
			Direct Effect	-0,113	-0.253; 0.018
			Total Indirect	-0,009	-0.094; 0.079
			**Indirect Effects Via**		
			None		
			**Predictor**	Estimate	Bootstrap 95% CI
			Goal Content Intrinsic -> Weight Change		
			Total Effect	0,008	-0.103; 0.111
			Direct Effect	0,050	-0.071; 0.156
			Total Indirect	-0,042	-0.098; 0.005
			**Indirect Effects Via**		
			Action Control	-0,020	-0.045; -0.002
			**Predictor**	Estimate	Bootstrap 95% CI

Goal Content Extrinsic -> Weight Change		
Total Effect	-0,074	-0.208; 0.042
Direct Effect	-0,099	-0.234; 0.041
Total Indirect	0,025	-0.020; 0.081
**Indirect Effects Via**		
None		

Maintainers

**Predictor**	Estimate	Bootstrap 95% CI
Climate Care -> Weight Change		
Total Effect	0,004	-0.021; 0.028
Direct Effect	0,007	-0.019; 0.033
Total Indirect	-0,003	-0.012; -0.005
**Indirect Effects Via**		
None		

**Predictor**	Estimate	Bootstrap 95% CI
Satisfaction Basic Psychological Needs -> Weight Change		
Total Effect	-0,049	-0.078; -0.024
Direct Effect	-0,045	-0.074; -0.018
Total Indirect	-0,004	-0.018; 0.009
**Indirect Effects Via**		
Eating Autonomous	0,009	0.001; 0.021

**Predictor**	Estimate	Bootstrap 95% CI
Goal Content Intrinsic -> Weight Change		
Total Effect	0,003	-0.022; 0.028
Direct Effect	-0,004	-0.031; 0.022
Total Indirect	-0,043	-0.004; 0.019

Predictor	Estimate	Bootstrap 95% CI
**Indirect Effects Via**		
Eating Autonomous	0,007	0.001; 0.016
Eating Controlled	0,002	0.000; 0.006
**Predictor**	Estimate	Bootstrap 95% CI
Goal Content Extrinsic -> Weight Change		
Total Effect	0,012	-0.010; 0.034
Direct Effect	0,007	-0.017; 0.030
Total Indirect	0,006	-0.003; 0.015
**Indirect Effects Via**		
None		

<u>Regainers</u>

**Predictor**	Estimate	Bootstrap 95% CI
Climate Care -> Weight Change		
Total Effect	-0,091	-0.179; -0.007
Direct Effect	-0,068	-0.151; 0.015
Total Indirect	-0,023	-0.063; 0.012
**Indirect Effects Via**		
Action Plan	-0,019	-0.047; -0.003
**Predictor**	Estimate	Bootstrap 95% CI
Satisfaction Basic Psychological Needs -> Weight Change		
Total Effect	-0,144	-0.211; -0.081
Direct Effect	-0,106	-0.179; -0.037
Total Indirect	-0,038	-0.080; -0.003
**Indirect Effects Via**		
Action Control	-0,015	-0.038; -0.003
Action Plan	-0,020	-0.045; -0.003
**Predictor**	Estimate	Bootstrap 95% CI
Goal Content Intrinsic -> Weight Change		

Total Effect	0,066	-0.015; 0.147
Direct Effect	0,109	0.026; 0.191
Total Indirect	-0,043	-0.079; -0.010
**Indirect Effects Via**		
Action Control	-0,017	-0.041; -0.004

**Predictor**	Estimate	Bootstrap 95% CI
Goal Content Extrinsic -> Weight Change		
Total Effect	0,004	-0.065; 0.074
Direct Effect	-0,028	-0.092; 0.037
Total Indirect	0,032	-0.001; 0.061
**Indirect Effects Via**		
Action Control	0,016	0.003; 0.035
Eating Controlled	0,017	0.001; 0.038
